# From across the Seas

**Published:** 1916-05-27

**Authors:** 


### FROM ACROSS THE SEAS.

### The Ideal Hospital Superintendent.

The superintendent may be a clergyman or a doctor or a layman, but he *must* be a remarkable man. He should be supreme over everyone in the house, including internes and nurses. As to the internes his authority is, of course, one of discipline, for in matters medical they must owe their responsibility to the attending staff. As to the nurses, their discipline is through the chief nurse, who, under the superintendent, represents the hospital authority over them. . . . The superintendent is expected to keep the peace with everyone *in* the hospital, and make peace with everyone *outside* it; and this is no sinecure, when the hospital is full and running behind financially, and a dozen influential people are demanding admission for free patients.—*The Hospital World* (Toronto).

### A Record Quotation.

Medical lecturers are fond of poetical quotations, but in point of length one used by Dr. W. N. Bradley, the retiring president of the Philadelphia Pediatric Society, as reported in *The Archives of Pediatrics*, New York, must score almost a record. The quotation consisted of thirteen four-line verses from Kipling's "Explorer," and was intended to illustrate the depth of sacrifice and suffering and the joy in attainment of exploration, and, translated to the realms of medicine, of research. Our English medical lecturers this year have been unusually sparing of quotations; with so promising a subject as "Medicine, Magic, and Religion." Dr. W. H. R. Rivers, in his two Fitzpatrick Lectures, contented himself with one quotation of a few lines of verse, while Dr. F. W. Mott, in giving the Lettsomian Lecture on the "Effects of High Explosives upon the Central Nervous System," an unsympathetic subject from the quotation point of view, drew upon both Byron and Shakespeare for illustration. We remember some years ago a lecturer on brain surgery making use of Clough's lines: "And not by eastern windows only, when daylight comes, comes in the light," the use of which in connection with his subject was ingenious.

